# Analysis of Prognostic Risk Factors for Ischemic Stroke in China: A Multicentre Retrospective Clinical Study; A National Survey in China

**DOI:** 10.2174/1567202619666220331160024

**Published:** 2022-07-26

**Authors:** Yuting Cao, Ying Chen, Xiaoli Zhang, Yongjun Wang

**Affiliations:** ^1^ Department of Obstetrics and Gynecology, Beijing Tiantan Hospital, Capital Medical University, Beijing, China;; ^2^ China National Clinical Research Center for Neurological Diseases, Beijing Tiantan Hospital, Capital Medical University, Beijing, China;; ^3^ Department of Neurology, Beijing Tiantan Hospital, Capital Medical University, Beijing, China

**Keywords:** Sex, ischaemic stroke, functional prognosis, stroke recurrence, mortality, public health

## Abstract

**Background:**

Stroke is a serious disease that threatens human health both in China and worldwide. Identifying and establishing its risk factors are prerequisites for intervention and evaluation of prognosis. Over the years, risk factors, such as age, diabetes, and hypertension, have gradually been established. However, at present, there is no consensus on the influence of sex on the prognosis of ischaemic stroke.

**Aims:**

The aims of our research was to analyse the correlation between sex and poststroke prognosis based on the results of the Third China National Stroke Registry [CNSR-III], as well as the influence of other risk factors that may be confounded by sex on ischaemic stroke and potential interventions.

**Methods:**

The CNSR-III recruited 14146 acute ischaemic stroke [AIS] patients between 2015 and 2018. Our study included 13,972 patients who had complete follow-up information. This research analysed basic information, socioeconomic status, lifestyle habits, medical history, and poststroke prognosis.

**Results:**

There was a conspicuous relationship between sex and functional prognosis, stroke recurrence and all-cause death due to ischemic stroke in univariate analysis. Male stroke patients had a better prognosis than female patients. In multivariate analysis, we found that age, atrial fibrillation [AF], diabetes, hypertension and the severity of stroke had adverse effects on ischemic stroke prognosis. After adjustment for other risk factors, the functional prognosis of female patients at 3 months was worse than that of male patients [odds ratio [OR] 1.16, 95% confidence interval [CI], 1.025-1.314]. Sex had a nonsignificant association with stroke recurrence at 3 months [hazard ratio [HR] 1.141, 95% CI, 0.975-1.336]. Furthermore, compared to male patients, female stroke patients had a lower cumulative death rate at 12 months [HR 0.777, 95% CI, 0.628-0.963].

**Conclusion:**

Our study identified sex differences in stroke-related disability, recurrence, and death and attempted to explain the causes of these differences. Our study clearly showed that a large proportion of this difference could be attributed to age, socioeconomic factors, lifestyle habits, and medical history, confounded by sex differences rather than sex per se.

## 1 INTRODUCTION

Stroke is the primary cause of death and restriction of daily activities in China [1]. Acute ischaemic stroke [AIS] and transient ischaemic attack [TIA] are the most common stroke types, accounting for approximately 70% of all strokes [2]. Due to the high incidence, recurrence, and mortality of stroke and the limitation of daily activities, stroke has placed a tremendous burden on patients, families, and even society [3]. In recent years, the effect of sex on poststroke prognosis has become a matter of concern.

Some previous studies suggested that sex was a predictor of poststroke prognosis and that females had a better prognosis than males [4-6], whereas other studies indicated that males had better prognostic outcomes [7, 8].

However, regarding the impact of sex on poststroke prognosis, recent studies have not reached an agreement. Increasing evidence has shown that sex differences in the prognosis of ischaemic stroke are caused by sex-related differences in age, comorbidities, and severity of stroke [9, 10].

The aim of our research was to analyse the correlation between sex and poststroke prognosis and whether this relationship was affected by other factors, such as age and past medical history. Understanding the underlying mechanisms of sex differences in stroke prognosis can lead to the identification of modifiable factors and interventions.

## 2 MATERIALS AND METHODS

All participants in our study were recruited from the Third China National Stroke Registry [CNSR-III]. A total of 14146 participants with AIS at 7 days from symptom onset were enrolled in the CNSR-III. In addition, 174 patients were excluded due to a lack of 3 months of follow-up information. A total of 13972 participants with AIS were enrolled in our study.

### 2.1 Baseline Data Collection

The baseline data included basic information [age, sex], socioeconomic factors [marital status, patients’ living arrangements before admission, education, health insurance, and family monthly income per capita], lifestyle habits [smoking, alcohol overuse], body mass index [BMI calculated as weight in kilograms divided by height in metres squared, kg/m^2^], medical history [coronary heart disease, hypertension, prior stroke, dyslipidaemia, diabetes, and atrial fibrillation], prestroke modified Rankin Scale [mRS score], aetiology classification of ischaemic stroke performed according to the Trial of ORG 10172 in Acute Stroke Treatment [TOAST] criteria and severity of stroke on admission [National Institutes of Health Stroke Scale, NIHSS].

### 2.2 Follow-up and Outcomes

Patients were followed up with face-to-face interviews at 3 months and telephonic interviews at 6 and 12 months by trained research coordinators based on a standardized interview protocol.

The poststroke prognosis referred to disability after stroke, stroke recurrence, and all-cause death. An mRS score of 3–5 was considered a poststroke disability. Recurrent stroke was defined as an exacerbation of the initial symptoms, new neurological symptoms lasting more than 24 hours, or readmission to the hospital after diagnosis of cerebral haemorrhage, ischaemic stroke, or subarachnoid haemorrhage. All-cause death was defined as the cumulative rate of death from any cause at 3, 6, and 12 months after stroke and was confirmed by a death certificate issued by the hospital or the local citizens' registry. Information regarding poststroke outcomes was required to be obtained at each follow-up.

### 2.3 Statistical Analysis

The baseline characteristics of women and men were compared using the chi-square test, as well as Fisher’s exact test when necessary. Variables with a *p*-value less than 0.05 were regarded as significant variables. We adjusted for these significant variables in multivariate regression analysis. Univariate and multivariate logistic regression analyses were performed, and an odds ratio [OR] with a 95% confidence interval [CI] was used to evaluate the relationship between sex and functional outcomes. The association of sex with stroke recurrence and death was assessed by univariate and multivariate Cox proportional hazard regression analyses, and hazard ratio [HR] with 95% CI was evaluated. All analyses were performed with SAS V.9.4 software. A two-sided *p-*value <0.05 was considered indicative of statistical significance.

## 3 RESULTS

### 3.1 Demographic Data and Risk Factors for Ischaemic Stroke

Male patients tended to be younger than female patients [mean age 61.17 *vs*. 64.72 years, *p*<0.000^1^]. Compared with male patients, female patients had higher BMI scores and were more likely to live alone, but the difference was not statistically significant. Women were less likely to be married [89.95% *vs*. 95.52%, *p*<0.000^1^] than men. Compared with men, women were more likely to have a primary school education or less [28.29% *vs*. 18.16%, *p*<0.000^1^], have a lower reimbursement rate for medical insurance [33.8% *vs*. 46.44%, *p*<0.000^1^], and earned less than 700 yuan a month [8.18% *vs*. 4.44%, *p*<0.000^1^].

**Fig. (1):**
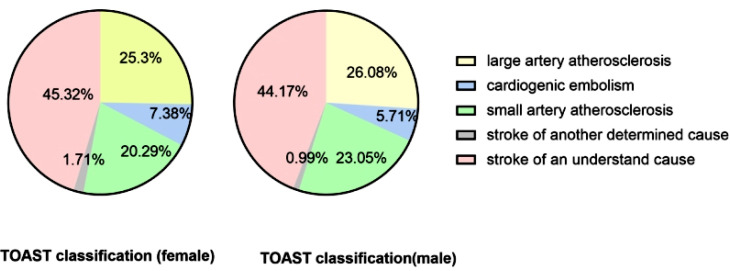
TOAST classification of female and male patients with ischaemic stroke.

Clear sex differences existed in stroke risk factors. Far more men than women had a history of smoking and alcohol overuse [44.58 *vs*. 3.7%, *p*<0.0001, 23.62% *vs*. 0.57%, *p*<0.000^1^]. In terms of comorbidities, more women had hypertension [68.35% *vs*. 60.29%, *p*<0.000^1^], diabetes [27.47% *vs*. 21.55%, *p*<0.000^1^], coronary heart diseases [13.14% *vs*. 9.3%, *p*<0.000^1^], atrial fibrillation [8.66% *vs*. 6.17%, *p*<0.000^1^], and women had a higher proportion of prestroke disability [5.14% *vs*. 4.18%, *p*=0.0107] than men. No sex differences were found concerning hyperlipidaemia or a history of stroke. Women had higher NIHSS scores [4.66 *vs*. 4.28, *p*<0.000^1^] and more cardiogenic stroke than men [7.38% *vs*. 5.71%, *p*=0.000^2^] (Table **1** and Fig. **1**). According to TOAST classification, male and female stroke patients were compared, respectively. In both male and female stroke patients, the proportion of stroke from an undetermined cause was the highest, followed by large artery atherosclerosis, followed by small artery atherosclerosis and cardiogenic embolism, and the proportion of stroke from another determined cause was the lowest.

Multivariate regression analysis was conducted to investigate the influence of factors other than sex on the prognosis of a stroke at 3 months Table **2**. Age, hypertension, diabetes, mRS score before onset, and NIHSS score on admission were independent risk factors for the functional prognosis of ischaemic stroke. However, cardiogenic stroke was a protective factor for the functional prognosis of ischaemic stroke. Coronary heart disease, diabetes, atrial fibrillation, and the NIHSS score on admission were closely related to the recurrence of stroke. Increased age, atrial fibrillation, coronary heart disease, and a higher NIHSS score on admission might increase stroke-related mortality.

### 3.2 Outcomes at 3, 6, and 12 Months After Ischaemic Stroke Onset

In univariate analysis, sex differences in the prognosis of recurrence, disability, and mortality at 3, 6, and 12 months after ischaemic stroke are illustrated in Figs. (**2** and **3**).

Fig. (**2**) reveals that the recurrence rate of stroke in female patients was higher than that in male patients at 3 months [7.27% *vs*. 6.18%, p=0.0164 6 months [8.84% *vs*. 7.75%, *p*=0.0297], and 12 months [10.72% *vs*. 9.71%, *p*=0.0685], but the difference was not statistically significant at 12 months.

Fig. (**2**) demonstrates the functional situation of male and female ischaemic stroke patients in different periods before and after stroke. Female patients had a worse functional prognosis than male patients at 3 months [OR 1.427, 95% CI, 1.294-1.572], 6 months [OR 1.394, 95% CI, 1.259-1.5^44^], and 12 months [OR 1.356, 95% CI, 1.226-1.^5^].

**Fig. (3):**
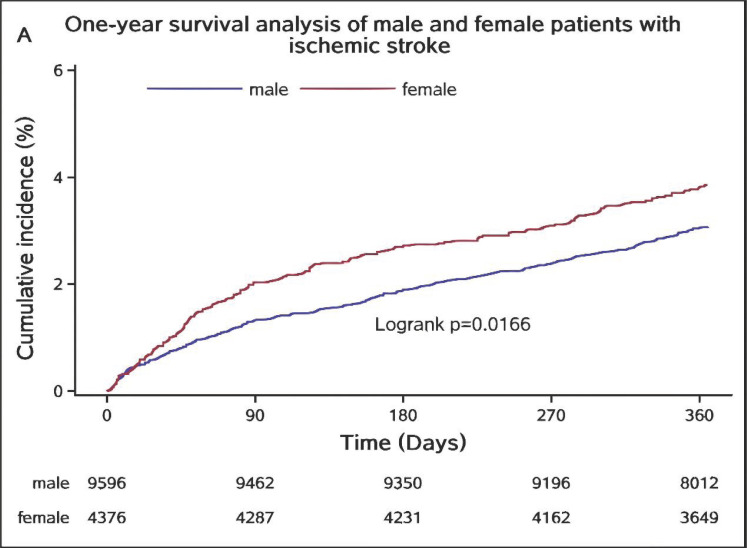
One-year survival analysis of male and female patients with ischemic stroke. The survival of male and female stroke patients was compared without considering other factors. The survival rate of female stroke patients was higher than that of male stroke patients at 3, 6, or 12 months after the onset of stroke, and there was statistical significance (p=0.0166).

The survival curve of patients with ischaemic stroke shows that the survival rate of women was significantly higher than that of men [p=0.0166] (Fig. **3**). Females had a better survival prognosis within 1 year than males.

After adjusting for other significant variables, we fully analysed sex differences in stroke outcomes at 3, 6, and 12 months Table **3**. The functional prognosis of women was still worse than that of men at 3 months after ischaemic stroke [OR 1.16, 95% CI, 1.025-1.314]. After adjustment, sex had a nonsignificant association with stroke recurrence [HR: 1.141, 95% CI, 0.975-1.336]. Women were more likely to survive than men at 12 months [HR 0.777, 95% CI, 0.628-0.963].

## 4 DISCUSSION

### 4.1 Sex and Stroke Prognosis

Univariate analysis suggested that women were more likely to be disabled after an ischaemic stroke. However, multivariate logistic regression analysis showed that women had a worse functional prognosis than men only at 3 months and that there were no differences in functional prognosis at 6 and 12 months. A consistent feature of these studies was that women had worse functional outcomes after stroke than men. One possible reason is that the same nerve damage affects men and women differently. Similarly, insular damage adversely affects functional outcomes in women but not in men [11]. Paolucci *et al*. pointed out the reason that men are more able to complete daily activities independently than women due to the difference in muscle strength between them [12]. More physical impairments and greater restrictions on daily activities are more likely to affect female stroke patients [13-15]. A study in Japan reported that after multivariate adjustment, women were less likely to have mRS scores of 0 to 1 [OR 0.802, 95% CI, 0.741-0.868, *p* =0.000^1^] and more likely to have mRS scores of 4 to 6 [OR 1.410, 95% CI, 1.293-1.537, *p*= 0.000^1^] than men [16]. In the International Stroke Trial, women had a much higher frequency of disability. The lack of a neuroprotective effect of oestrogen in these predominantly postmenopausal women might be a factor [8]. However, Renoux *et al*. suggested that sex was not associated with an increased risk of a higher mRS score, whether one month after the stroke or 5 years after the stroke [17].

We found that females had a higher rate of stroke recurrence at 3 months, 6 months, and 12 months of follow-up, but the *p-*value at 12 months was not statistically significant in univariate analysis. However, Cox multivariate analysis found no significant connection between sex and stroke recurrence. The preadjustment analysis, the Cox analysis containing risk factors, and the subgroup analysis by age did not find any relationship between sex and recurrence of stroke. Sex had a nonsignificant association with stroke recurrence even in the perimenopausal age groups [18]. However, Jiann-Der Lee indicated that men showed an increase in the risk of one-year stroke recurrence in multivariate logistic regression analysis [4]. Our study added to the evidence that sex was not associated with stroke recurrence, rather than those men have a higher recurrence rate.

As shown in the survival curve, the survival rate of female patients with ischaemic stroke was significantly higher than that of male patients at 3 months, 6 months, and 12 months. Multivariate analysis showed that women had a lower mortality rate than men, only at 12 months. Previous studies have presented different perspectives on the survival outcomes of stroke patients of different sexes. Christel Renoux pointed out that the apparent higher mortality in women was no longer seen when controlling for age [HR 0.95, 95% CI, 0.84–1.0^7^] and comorbidities and severity of stroke [HR 0.82, 95% CI, 0.72–0.94] [17]. Reeves *et al*. revealed that sex differences in stroke deaths were strongly modified by age. Under the age of 45 years, mortality rates for male and female stroke patients were almost the same. Between 45 and 74 years old, the mortality rate of female stroke patients was substantially lower than that of males. When the age was over 85 years, the female mortality rate was higher than that of males [19]. A Danish study found that stroke affected women and men differently because of sexual dimorphism in stroke, with differences observed both in the clinic and the laboratory. The stroke affected older women more severely than it did older men. The results showed an innate female superiority in the likelihood of survival after stroke [10]. Research by Carcel *et al*. supported our view and confirmed the sex differences in disability and death after stroke. They suggested that in-hospital management and the use of preventative medication may contribute to sex differences in outcome [20]. In the future, we can further extend our study to analyse sex differences in treatment during hospitalization and prophylactic medication after discharge and their association with stroke outcomes.

### 4.2 Other Risk Factors and Stroke Prognosis

Previous studies have proposed adverse effects of age on functional prognosis and stroke-related death, which was also confirmed in our study [21, 22]. Harris *et al*. found that the risk of stroke-related disability and death increased by 1.33 times for each year the patient’s age increased [23]. Animal experiments showed that young rats' nerve cell and glial cell repairability and brain regeneration ability were better than old rats [24, 25]. Perhaps in future studies, we can stratify by age to further clarify sex differences in the prognosis of disability, recurrence, and mortality after stroke. Our study found that hypertension was associated with the prognosis of stroke-related disability, and other studies also found that hypertension increased the recurrence and mortality rate of stroke. When diastolic blood pressure increased by 10 mmHg or systolic blood pressure increased by 10 mmHg, the mortality rate of ischaemic stroke showed a corresponding increase of 1.36 times [95% CI, 1.29-1.^43^] and 1.19 times [95% CI, 1.16-1.^23^], respectively [26]. For every 10 mmHg increase in systolic blood pressure, the stroke recurrence rate increased by 4.2%. One possible explanation is that hypertension increases early stroke recurrence within 14 days, which, in turn, increases disability, recurrence, and death from stroke [27]. In our study, diabetes was found to have an adverse effect on disability and recurrence of stroke. Our results have been explained in previous animal models. In a middle cerebral artery occlusion animal model, a hyperglycaemic environment aggravated cerebral ischaemia and reperfusion injury, further leading to a poor prognosis of stroke [28, 29]. Furthermore, there were sex differences in the impact of diabetes on stroke, and it had a more severe impact on women [30]. Previous studies only proved that diabetes and hyperglycemia at admission are risk factors for the prognosis of stroke but failed to clarify the specific relationship between blood glucose levels and the prognosis of stroke. The study conducted by Nan Dong *et al.* classified patients according to their glycated hemoglobin (HbA1c) at baseline at admission and found that the prognosis of patients in the HbA1c group below 5% was significantly better than that in the HbA1c group above 8%. Elevated HbA1c (as a continuous variable) was associated with poor functional outcomes in stroke [31].

Our study clearly suggested that heart disease and atrial fibrillation [AF] lead to more recurrent strokes and deaths. This was also confirmed in a previous study, in which stroke patients in Poland not only had worse functional outcomes than those in the United States but also had a threefold higher mortality rate. The reason was that a large proportion of Polish stroke patients had heart disease, which leads to more severe stroke symptoms and higher mortality [32]. AF-related cardioembolic stroke, which causes sudden occlusion of large cerebral arteries without sufficient collateral blood flow, results in more severe strokes [33]. Some early research suggested that the impact of AF on the poor prognosis of stroke was due to the severe neurological deficits and older age of patients with AF [34, 35]. As our research indicated, women present with more severe stroke symptoms, as measured by the NIHSS, and stroke severity could affect the overall poor prognosis of patients with stroke [36, 37]. Specifically, Jain *et al*. suggested that for each one-point increase in the stroke scale at baseline, there was a 2.3-fold increased likelihood of mortality and a 3-fold increased likelihood of functional disability [38]. [Cardioembolic stroke is generally the most severe ischaemic stroke subtype, as it is associated with poor functional outcomes at hospital discharge, early and long-term recurrence and higher mortality] [39, 40]. Cardioembolic stroke involves a larger embolization area than other types of stroke [41]. In our study, we did not find that the prognosis of cardioembolic stroke was worse than that of other types of stroke or even better in terms of functional prognosis. There might be a bias in patient selection, which requires further stratified analysis.

Previous studies focused on the impact of some common diseases on the prognosis of stroke but ignored the psychological and mental state of patients that may have a certain impact on the prognosis of stroke. Depressive symptoms at baseline significantly increased the risk of stroke [HR 1.31,95%CI: 1.18-1.^44^] and stroke mortality [HR 1.29,95%CI: 1.03-1.61] [42]. One possible mechanism is that depressive symptoms can affect a patient's lifestyle, such as physical inactivity, obesity and poor treatment compliance [43]. In a multicenter observational cohort study conducted in South Korea, a significant correlation was found between the onset time of stroke and the severity and prognosis of stroke. Stroke onset was classified into day-onset (6:00-18:00) and night-onset (18:00-6:00). It was found that patients with night-onset stroke had more severe clinical symptoms and were more likely to experience early neurological deterioration within 72 hours of onset, with poorer functional outcomes 3 months later. The severity and progression of stroke had circadian variation [44]. In addition, numerous studies have confirmed that the level of inflammatory cytokines in patients is also closely related to the prognosis after stroke. In a recent cross-sectional study, circulating tumor necrosis factor-stimulating gene-6 (TSG-6) was revealed for the first time as an independent predictor of adverse outcomes in non-cardioembolic stroke. Elevated levels of TSG -6 were positively associated with poor functional outcomes in non-cardioembolic stroke. An elevated TSG-6-to-interleukin-8 ratio might suggest a favorable outcome at 3 months [45].

### 4.3 Strengths and Limitations

Our study confirmed that women had a worse functional prognosis at 3 months and that men had a worse survival prognosis at 12 months. Sex had a nonsignificant association with stroke recurrence. Our research analysis included only common classic risk factors but did not include some specific risk factors that might be related to the prognosis of stroke, including pregnancy, delivery, childbirth, puerperium, oral contraceptives, and hormone replacement therapy after menopause. We followed up with patients by telephone and not by face-to-face interview because too many

Patients were enrolled in this large registry. We could not perform Cox proportional hazard regression due to the lack of precise follow-up data about the death or stroke recurrence time of patients during telephone follow-ups to identify the correlation between the prognostic factors and time. The follow-up was short, and we could not further understand the prognosis of stroke. In the future, we plan to extend the follow-up time to explore the association between sex and longer-term stroke outcomes. In future studies, the psychological and mental state of stroke patients, onset time of stroke, and menstrual and reproductive history of female patients can be further included in the baseline to understand the difference between patients with stroke of different genders at baseline and its impact on the prognosis of stroke. The blood biochemical index results of stroke patients were not included in our study, and the differences in admission blood glucose, glycosylated hemoglobin, blood lipids, coagulation factors, TSG-6 and other inflammatory factors among patients of different genders and their influence on the prognosis of stroke can be included in future studies. In addition, different treatment methods also have different effects on the prognosis of stroke. Future studies should focus on whether there are differences in the treatment choice of patients of different genders and further explore the impact of these differences on the prognosis of stroke.

## CONCLUSION

As mentioned above, we found that female stroke patients had worse functional prognosis than male patients; however, women were not significantly different from men regarding stroke recurrence and death and, after adjustment for other significant variables, they were found to be even better than men. The onset age of ischaemic stroke, more comorbidities, and the severity of stroke symptoms made comprehensive contributions to the sex differences in stroke prognosis rather than sex per se. Our research concluded that age, diabetes, coronary heart disease, hypertension, atrial fibrillation, stroke severity, and TOAST classification were all independent predictors of stroke prognosis. Active and reasonable control of diabetes, hypertension, coronary heart disease, atrial fibrillation, and other complications would improve prognosis.

## Figures and Tables

**Fig. (2):**
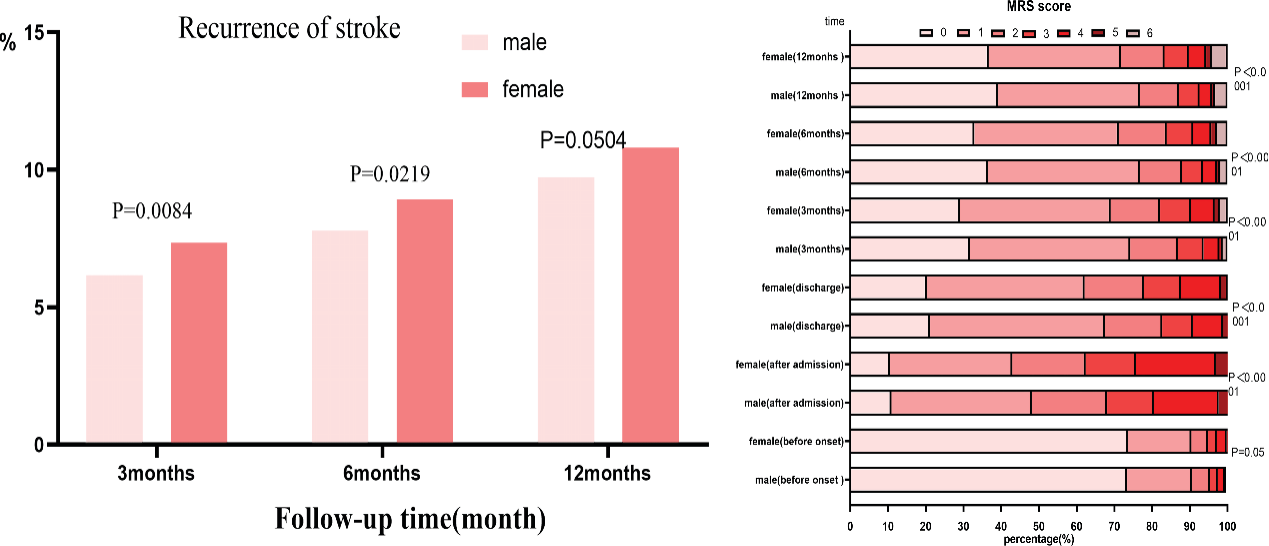
Stroke recurrence at 3 months, 6 months, and 12 months after ischemic stroke and the mRS scores of patients with ischemic stroke in different periods. Follow-up at 3, 6, and 12 months after the onset of stroke showed that the recurrence rate of stroke in female patients was higher than that in male patients, and only the recurrence rate at 3 and 6 months was statistically significant (p=0.0164; p=0.0297), while the recurrence rate at 12 months was not (p=0.0685). The mRS scores were performed for all stroke patients at onset, admission, discharge, 3, 6, and 12 months after onset. The disability rate (mRS: 3-5) of female patients was higher than that of male patients at 3, 6, and 12 months after stroke, and the difference was statistically significant.

**Table 1:** Demographic data and risk factors for ischaemic stroke.

**Basic Features**	**Total** **n=13972**	**Male** **n=9596**	**Female** **n=4376**	***P-Value***
Average age (years), mean	62.28	61.17	64.72	<0.0001
Body mass index, kg/m^2^	24.69	24.67	24.74	0.5714
Marital status, n (%)	-	-	-	<0.0001
Unmarried	88 (0.63%)	82 (0.85%)	6 (0.14%)	-
Married	13102 (93.77%)	9166 (95.52%)	3936 (89.95%)	-
Other	755 (5.4%)	332 (3.46%)	423 (9.67%)	-
Unknown	27 (0.19%)	16 (0.17%)	11 (0.25%)	-
Living condition, n (%)	-	-	-	0.082
Living alone	706 (5.05%)	464 (4.84%)	242 (5.53%)	-
Living with others	13266 (94.95%)	9132 (95.16%)	4134 (94.47%)	-
Education level, n (%)	-	-	-	<0.0001
Elementary school or below	2981 (21.34%)	1743 (18.16%)	1238 (28.29%)	-
Middle school	4074 (29.16%)	3012 (31.39%)	1062 (24.27%)	-
High school or above	3866 (27.67%)	3110 (32.41%)	756 (17.28%)	-
Unknown	3051 (21.84%)	1731 (18.04%)	1320 (30.16%)	-
Insurance schemes, n (%)	-	-	-	<0.0001
Medical insurance for urban worker and Public health services	5935 (42.48%)	4456 (46.44%)	1479 (33.8%)	-
Urban residents' basic medical insurance and Rural cooperation medical insurance	7146 (51.15%)	4494 (46.83%)	2652 (60.6%)	-
Self-payment	853 (6.11%)	620 (6.46%)	233 (5.32%)	-
Other	38 (0.27%)	26 (0.27%)	12 (0.27%)	-
Family monthly income per capita, n (%)	-	-	-	<0.0001
<700 yuan	784 (5.61%)	426 (4.44%)	358 (8.18%)	-
700~2300 yuan	4947 (35.41%)	3244 (33.81%)	1703 (38.92%)	-
>2300 yuan	4752 (34.01%)	3547 (36.96%)	1205 (27.54%)	-
Unknown	3489 (24.97%)	2379 (24.79%)	1110 (25.37%)	-
Current smoker, n (%)	4440 (31.78%)	4278 (44.58%)	162 (3.7%)	<0.0001
Alcohol overuse, n (%)	2292 (16.4%)	2267 (23.62%)	25 (0.57%)	<0.0001
Medical history, n (%)	-	-	-	-
Hypertension	8776 (62.81%)	5785 (60.29%)	2991 (68.35%)	<0.0001
Diabetes mellitus	3270 (23.4%)	2068 (21.55%)	1202 (27.47%)	<0.0001
Lipid metabolism disorders	1067 (7.64%)	740 (7.71%)	327 (7.47%)	0.6218
Stroke	3082 (22.06%)	2159 (22.5%)	923 (21.09%)	0.0629
Coronary heart diseases	1467 (10.5%)	892 (9.3%)	575 (13.14%)	0.0001
Atrial fibrillation	971 (6.95%)	592 (6.17%)	379 (8.66%)	0.0001
mRS score (before onset), n (%)	-	-	-	0.0107
0-2	13346 (95.52%)	9195 (95.82%)	4151 (94.86%)	-
3-5	626 (4.48%)	401 (4.18%)	225 (5.14%)	-
NIHSS score on admission, mean	4.4	4.28	4.66	<0.0001
Aetiology according to TOAST classification	-	-	-	0.0002
Cardiogenic embolism, n (%)	871 (6.23%)	548 (5.71%)	323 (7.38%)	-
Other (large artery atherosclerosis, small artery occlusion, stroke of another determined cause, stroke of an undetermined cause), n (%)	13101 (93.77%)	9048 (94.29%)	4053 (92.62%)	-

Alcohol overuse was defined as ≥2 standard alcohol beverages/per day;

mRS: Modified Ranking Scale;

NIHSS: National Institutes of Health Stroke Scale, used to describe the severity of stroke;

TOAST: Trial of ORG 10172 in Acute Stroke Treatment.

**Table 2:** Multivariate logistic regression analysis for other risk factors and outcomes at 3 months after ischaemic stroke onset.

**Variables**	**Disability OR (95% CI)**	**Recurrence OR (95% CI)**	**Mortality OR (95% CI)**
Age	1.034 (1.029-1.04)	1.006 (0.999-1.013)	1.057 (1.042-1.073)
Marital status	-	-	-
Married	0.457 (0.247-0.847)	1.225 (0.456-3.29)	0.747 (0.104-5.373)
Other	0.531 (0.278-1.015)	1.429 (0.515-3.967)	0.906 (0.121-6.774)
Unknown	0.409 (0.096-1.736)	0.693 (0.077-6.215)	0 (0-1.72)
Education level	-	-	-
Middle school	0.886 (0.76-1.032)	0.909 (0.749-1.102)	0.986 (0.662-1.468)
High school or above	0.963 (0.818-1.135)	1.022 (0.834-1.253)	0.974 (0.631-1.504)
Unknown	1.072 (0.916-1.255)	1.027 (0.836-1.262)	1.26 (.86-1.845)
Insurance schemes	-	-	-
Urban residents' basic medical insurance and Rural cooperation medical insurance	1.101 (0.973-1.246)	0.87 (0.747-1.012)	1.139 (0.826-1.572)
Self-payment	1.122 (0.894-1.408)	0.761 (0.557-1.04)	1.356 (0.775-2.373)
Other	1.39 (0.5-3.863)	0.778 (0.194-3.125)	3.033 (0.419-21.945)
Family monthly income per capita	-	-	-
700~2300 yuan	0.932 (0.741-1.172)	1.1 (0.806-1.503)	1.152 (0.643-2.062)
>2300 yuan	1.043 (0.821-1.326)	1.028 (0.743-1.422)	1.196 (0.647-2.21)
Unknown	1.041 (0.821-1.321)	1.041 (0.753-1.439)	1.022 (0.559-1.869)
Current smoker	0.896 (0.784-1.023)	0.919 (0.781-1.081)	0.879 (0.604-1.281)
Alcohol overuse	1.003 (0.851-1.182)	1.171 (0.967-1.419)	0.808 (0.484-1.348)
Medical history	-	-	-
Hypertension	1.12 (1.001-1.252)	1.053 (0.916-1.21)	0.792 (0.598-1.048)
Diabetes mellitus	1.217 (1.078-1.374)	1.239 (1.069-1.437)	1.364 (0.997-1.867)
Ischaemic stroke	-	-	-
Coronary heart diseases	0.961 (0.815-1.133)	1.216 (1.001-1.476)	1.529 (1.084-2.157)
Atrial fibrillation	1.132 (0.897-1.43)	1.452 (1.094-1.926)	2.142 (1.438-3.188)
mRS score (before onset)	1.962 (1.61-2.39)	0.84 (0.61-1.156)	1.251 (0.806-1.943)
NIHSS score on admission	1.252 (1.236-1.267)	1.037 (1.023-1.051)	1.121 (1.102-1.141)
Cardiogenic embolism	0.748 (0.579-0.966)	0.741 (0.535-1.027)	0.635 (0.391-1.031)

Marital status: Coefficients presented for marital status (unmarried as reference) with 95% CI.

Education level: Coefficients presented for education level (elementary or below as reference) with 95% CI.

Insurance schemes: Coefficients presented for insurance schemes (medical insurance for urban workers and public health services as a reference) with 95% CIs.

Family monthly income per capita: Coefficients presented for family monthly income per capita level (less than 700 yuan as reference) with 95% CI.

Cardiogenic embolism: Coefficients presented for cardiogenic embolism (other [large artery atherosclerosis, small artery occlusion, stroke of another determined cause, stroke of an undetermined cause] as reference) with 95% CI.

**Table 3:** Unadjusted and adjusted ORs or HRs of outcomes at 3, 6 and 12 months.

**-**	**Unadjusted** **(3months)**	**Adjusted** **(3months)**	**Unadjusted** **(6months)**	**Adjusted** **(6month)**	**Unadjusted** **(12months)**	**Adjusted** **(12month)**
Disability	1.427 (1.294-1.572)	1.16 (1.025-1.314)	1.394 (1.259-1.544)	1.023 (0.9-1.163)	1.356 (1.226-1.5)	0.956 (0.842-1.085)
Recurrence	1.184 (1.033-1.357)	1.141 (0.975-1.336)	1.151 (1.018-1.301)	1.127 (0.978-1.299)	1.115 (0.998-1.246)	1.061 (0.934-1.204)
Mortality	1.529 (1.167-2.004)	0.94 (0.692-1.278)	1.44 (1.143-1.814)	0.912 (0.702-1.186)	1.261 (1.043-1.525)	0.777 (0.628-0.963)

## Data Availability

The raw data supporting the conclusion of this article will be made available by the authors without undue reservation.

## LIST OF ABBREVIATIONS

AF: Atrial Fibrillation
AIS: Acute ischaemic Stroke
BMI: Body Mass Index
CI: Confidence Interval
CNSR-III: The Third China National Stroke
HR: Hazard Ratio
mRS: Modified Rankin Scale
NIHSS: National Institutes of Health
OR: Odds Ratio Registry Stroke Scale Stroke Treatment
TIA: Transient Ischaemic Attack
TOAST: Trial of ORG 10172 in Acute

## STANDARDS OF REPORTING

STROBE guidelines were followed.
